# State-of-the-art therapeutic strategies for targeting cancer stem cells in prostate cancer

**DOI:** 10.3389/fonc.2023.1059441

**Published:** 2023-03-09

**Authors:** Saravanan Ramesh, Preethi Selvakumar, Mohamed Yazeer Ameer, Sen Lian, Abdulqadir Ismail M. Abdullah Alzarooni, Shreesh Ojha, Anshuman Mishra, Ashutosh Tiwari, Ajeet Kaushik, Young Do Jung, Salem Chouaib, Vinoth-Kumar Lakshmanan

**Affiliations:** ^1^ Prostate Cancer Biomarker Laboratory, Faculty of Clinical Research, Sri Ramachandra Institute of Higher Education and Research, Chennai, India; ^2^ Department of Biochemistry and Molecular Biology, School of Basic Medical Sciences, Southern Medical University, Guangzhou, Guangdong, China; ^3^ Department of Urology, H.H. Sheikh Khalifa General Hospital, Al Salama, Opp. Ministry of Community Development, Umm Al Quwain, United Arab Emirates; ^4^ Department of Pharmacology and Therapeutics, College of Medicine and Health Sciences, United Arab Emirates University, Al Ain, United Arab Emirates; ^5^ Translational Research & Sustainable Healthcare Management, Institute of Advanced Materials, IAAM, Ulrika, Sweden; ^6^ NanoBioTech Laboratory, Department of Environmental Engineering, Florida Polytechnic University, Lakeland, FL, United States; ^7^ School of Engineering, University of Petroleum and Energy Studies (UPES), Dehradun, India; ^8^ Department of Biochemistry, Chonnam National University Medical School, Gwangju, Republic of Korea; ^9^ Thumbay Research Institute for Precision Medicine, Gulf Medical University, Ajman, United Arab Emirates; ^10^ INSERM UMR1186, Integrative Tumor Immunology and Genetic Oncology, Gustave Roussy, Equipe Labellisée par la Ligue Contre le Cancer, EPHE, Faculté de Médecine, Université Paris-Sud, Université Paris-Saclay, Villejuif, France

**Keywords:** prostate cancer, cancer stem cells, immunotherapy, chemotherapy, CRISPR, nanotechnology, photothermal ablation therapy

## Abstract

The development of new therapeutic strategies is on the increase for prostate cancer stem cells, owing to current standardized therapies for prostate cancer, including chemotherapy, androgen deprivation therapy (ADT), radiotherapy, and surgery, often failing because of tumor relapse ability. Ultimately, tumor relapse develops into advanced castration-resistant prostate cancer (CRPC), which becomes an irreversible and systemic disease. Hence, early identification of the intracellular components and molecular networks that promote prostate cancer is crucial for disease management and therapeutic intervention. One of the potential therapeutic methods for aggressive prostate cancer is to target prostate cancer stem cells (PCSCs), which appear to be a primary focal point of cancer metastasis and recurrence and are resistant to standardized therapies. PCSCs have also been documented to play a major role in regulating tumorigenesis, sphere formation, and the metastasis ability of prostate cancer with their stemness features. Therefore, the current review highlights the origin and identification of PCSCs and their role in anti-androgen resistance, as well as stemness-related signaling pathways. In addition, the review focuses on the current advanced therapeutic strategies for targeting PCSCs that are helping to prevent prostate cancer initiation and progression, such as microRNAs (miRNAs), nanotechnology, chemotherapy, immunotherapy, the clustered regularly interspaced short palindromic repeats (CRISPR)/CRISPR-associated protein 9 (Cas9) gene-editing system, and photothermal ablation (PTA) therapy.

## 1 Introduction

Prostate cancer is a complex disease that affects millions of men globally, predominantly in regions that rank highly on the Human Development Index (1). The identification and molecular characterization of genes associated with inherited susceptibility to prostate cancer and of genes in prostate cancer cells that tend to have somatic alterations suggest that infection or inflammation of the prostate contributes to the development of prostate cancer (2). In spite of various therapies, such as radiopharmaceutical therapy, hormonal therapy, chemotherapy, and immunotherapy, Lang et al. proposed that there is evidence that solid tumors originate from undifferentiated stem cell-like cells, which coexist within a heterogeneous tumor mass, that drive tumor formation, maintain tumor homeostasis, and initiate metastases (3). In addition, cancer cells display features of normal tissue organization, whereas cancer stem cells (CSCs) can drive tumor growth, evolution of the cancer stem cells model, and genetics proposed by Kreso et al. to describe the role of genetic diversity and non-genetic influences in contributing to tumor heterogeneity (4). The exocrine gland of prostatic ducts is lined with secretory luminal cells, rare neuroendocrine cells, and basal cells (5). Interestingly, basal cells in the prostate gland are intrinsically enriched in gene sets that are usually associated with stem cells, neurogenesis, and ribosomal RNA biogenesis (6). With regard to theragnosis, prostate cancer stem cells (PCSCs) are the driving force in the tumorigenesis, relapse, metastasis, and therapeutic resistance of prostate cancer (7). Clarke et al. recently proposed that DNA methylation and chromatin modification dictate the difference between stem cells and other types of cells. One part of the polycomb repressor complex 1 (PRC1), a chromatin modifier known as BMI1, usually mediates gene silencing by regulating chromatin structure required for self-renewal of hematopoietic stem cells, as well as for self-renewal of prostate gland stem cells. Collectively, epigenetic regulators, transcription factors, and miRNAs play very important roles in the self-renewal of stem cells (8, 9).

Survival of PCSCs could promote tumor initiation, migration, and relapse, and induce therapy resistance (10, 11). In addition, PCSC plasticity is anticipated to play a pivotal role in bone metastases of prostate cancer (12). PCSCs can be identified with cell surface markers expressed, and studies suggest that CD44 harbors them to exhibit tumor invasive properties (13, 14). Extensive endogenous signaling pathways and wide-ranging epigenetic modifications are involved in the maintenance of the stemness features of PCSCs, such as the wingless-related integration site (Wnt)/β-catenin, phosphoinositide 3-kinase (PI3K)/AKT, sonic hedgehog (SHH), Notch, and androgen receptor (AR) signaling pathways, and histone modifications (15). Accordingly, targeting these pathways to eradicate PCSCs is believed to have potential therapeutic value in prostate cancer therapy. Recently, Han et al. suggested that AR blockade induces reprogramming to a stem-like state characterized by hybrid epithelial and mesenchymal (E/M) traits and clinical aggressivity. Moreover, drug-persistent mesenchymal and stem-like prostate cancer cells are rescued by exogenous recombinant human neuregulin-b1 (NRG1) generating metastases (16). In this review, we briefly describe the origin and the identification of PCSCs as well as their specific stemness properties and processes associated with signaling pathways. Furthermore, we review the current emerging therapeutic strategies for targeting PCSCs, such as miRNAs, nanotechnology, chemotherapy, immunotherapy, the clustered regularly interspaced short palindromic repeats (CRISPR)/CRISPR-associated protein 9 (Cas9) system, and photothermal ablation (PTA). These distinctive strategies could impede prostate cancer initiation and development *via* targeting PCSCs’ stemness-associated signaling pathways.

## 2 Origin and identification of PCSCs

The origin of PCSCs is still subject to debate. Prostate stem cells (PSCs) are found in the basal and luminal layers, and gain mutations in tumor oncogene and suppressor gene led to become PCSCs during carcinogenesis (17). An adult PSC resembles the same transcriptional landscape of aggressive prostate cancer phenotype (18). Many research studies have demonstrated that PCSCs can originate from differentiated epithelial cells *via* epithelial–mesenchymal transition (EMT), in which epithelial cells gain the invasion and migration properties of stem cells through various molecular mechanisms and epigenetic modification. *In vitro* study has implied that the transition of the EMT phenotype to prostate cancer cells exhibits a high level of clone- and sphere-forming capability (19). Lee et al.’s experimental data showed that the reduction of DNA methyltransferase 1 (*DNMT1*) expression with 5-azacytidine treatment, a global demethylation agent, could induce EMT, and the cancer stem cell phenotype in prostate cancer cells and in turn promote cancer metastasis (20). Kong et al. documented that inducing a high level of expression of platelet-derived growth factor D in prostate cancer cells promotes the EMT phenotype *via* activating nuclear factor kappa B (NF-κB) signaling and the mammalian target of rapamycin kinase (mTOR) (21). Talati et al.’s study revealed that Janus kinase 2 (JAK2) and the signal transducers and activators of transcription 5 (STAT5) A/B (JAK2-STAT5A/B) signaling pathway is involved in promoting the EMT phenotype and expression of the cancer stem cell maker BMI1 in prostate cancer cells. Knockdown of Twist1, an EMT marker, in prostate cancer cells, resulted in the suppression of the STAT5A/B signaling that can induce EMT phenotype and sphere formation ability in prostate cancer (22).

Furthermore, *PTEN* (phosphatase and tensin homolog deleted on chromosome 10) is a tumor suppressor gene, and the deletion of *PTEN* in prostate cancer murine models leads to expansion of the PSC subpopulation and then tumor initiation (23). In addition, loss of the *PTEN* gene has been involved in promoting the transformation of prostate epithelial cells to the EMT phenotype (24). Estrogen receptors α and β (i.e., ERα and ERβ) play a differential role in prostate cancer development: overexpression of ERα cuases the tumor oncogene effect and, in contrast, overexpression of ERβ could induce the tumor suppressor effect (25, 26). Recent research has clarified the underlying molecular mechanism between ERβ and prostate tumorigenesis. *PTEN* deletion causes BMI1 activation, which is a critical regulator of PSCs’ self-renewal and malignant transformation, which in turn suppresses ERβ expression. Consequently, the suppression of ERβ has been implicated in prostate tumorigenesis initiation (27). *In vitro* study revealed that ERα promotes the EMT process of PCSCs *via* activating the NOTCH1 signaling pathway, which was mediated by the enhancer of Zeste homolog 2 (EZH2) (28).

In cancer therapy, cell surface markers could be used as paradigms for the specific identification and targeting of cancer cells as a means to eradicate them. The study by Collins et al. revealed that a population of CD44^+^/α2β1^high^/CD133^+^ cells can be isolated from prostate cancer patients. This population of cells has a high capacity to be self-renewable and to be able to proliferate (29). Furthermore, CD44^+^ PCSCs exhibited higher levels of cell proliferation, tumorigenesis, and metastasis potential than CD44^–^ PCSCs (13). Hurt et al. reported that CD44^+^- and CD24^–^enriched cells expressed stem cell-related transcription factors, such as BMI1 and octamer-binding transcription factor 3/4 (OCT-3/4), and exhibited *in vitro* prostasphere cell formation (30). The activity of the enzyme aldehyde dehydrogenase (ALDH) was increased in prostate cancer cells. The enzyme was also involved in intracellular retinoic acid synthesis, which is associated with prostate cancer development, and metastasis formation (31). Li et al. found that ALDH1A1 is a potential marker for malignant PSCs (32). Therefore, ALDH acted as a potential marker for identifying PCSC populations. Trerotola et al. revealed that cancer cell populations with CD133, trophoblast cell surface antigen 2 (TROP-2), and α2β1 integrin surface receptors displayed a stem cell phenotype and served as the ideal markers to characterize PCSCs (33). Sui et al. reported that a PC-3 and a DU145 cell line with a CD51 population exhibited high levels of sphere formation and metastatic initiation, and enhanced transcription factors Nanog and SRY-box transcription factor 2 (SOX2), compared with the CD51^–^ population. Therefore, CD51 acts as a novel surface marker to identify PCSCs (34). An *in vitro* and *in vivo* study demonstrated that CD54 contributed to the self-renewal and tumorigenesis of a PC-3 cell line. Knockdown of CD54 by short hairpin RNAs (shRNAs) attenuated tumorigenesis in a PC-3 cell line and demonstrated an increase in the survival period for mouse prostate xenograft models (35).

## 3 Stemness-related signaling pathways

**Figure 1:**
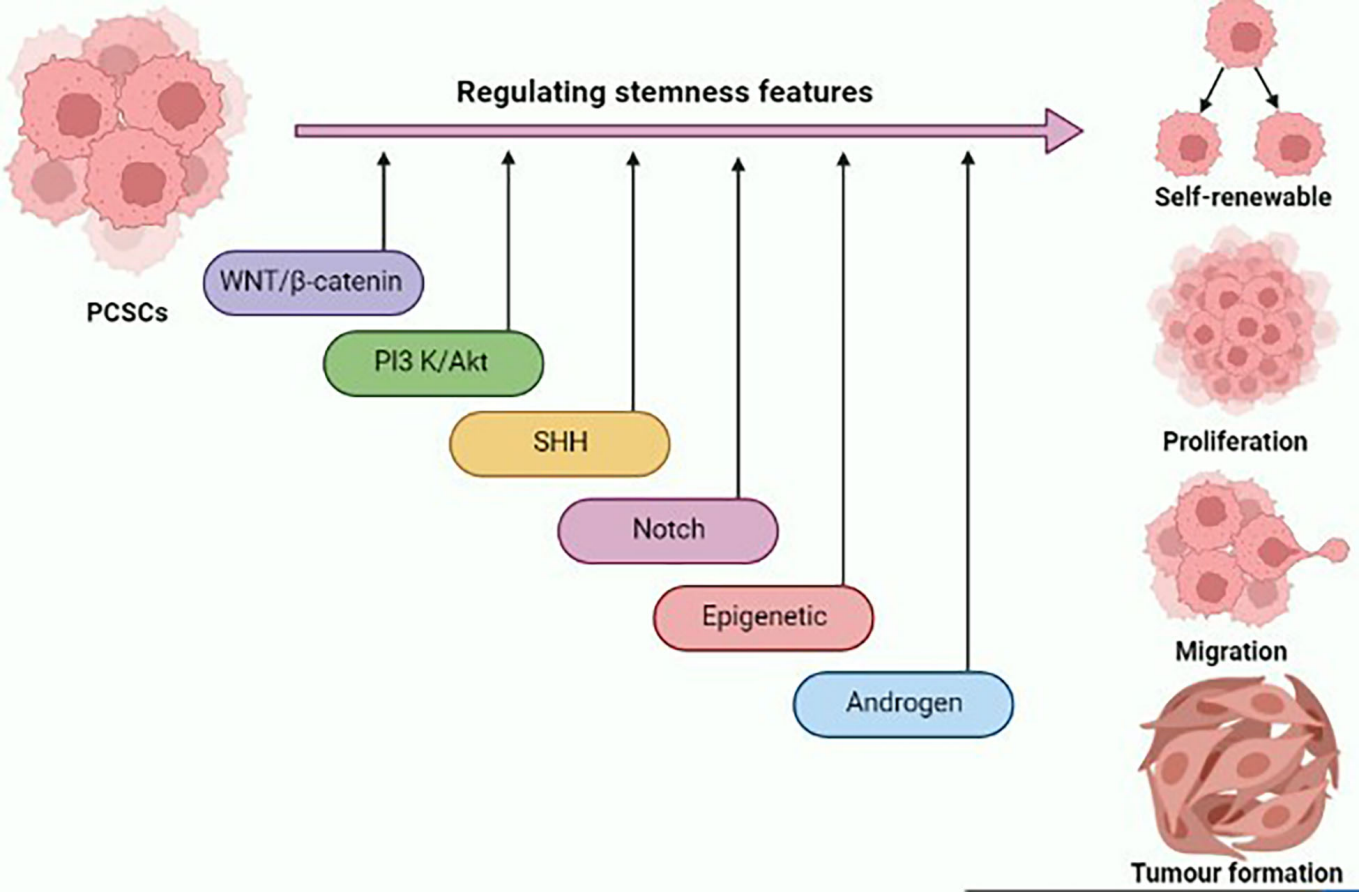
Graphical illustration representing the various signaling pathways and epigenetic landscape contributing to the regulation of stemness features of prostate cancer stem cells (PCSCs) such as self-renewal, proliferation, migration, and tumor formation. This figure was created with biorender.com.

Stem-like traits of PCSCs have been implicated in prostate cancer progression and metastasis formation. Deciphering the molecular network underlying the stem-like signature will provide novel insights for the development of potential therapeutic interventions for prostate cancer (**Figure 1**). Several signal transduction cascades are thought to contribute to the regulation of stemness properties of PCSCs, such as Wnt/β-catenin, PI3K/AKT, SHH, Notch, and AR signaling pathways. The Wnt pathway plays a critical role in the development process during the embryonic period, and it regulates adult stem cell self-renewal and differentiation (36). An *in vitro* study highlighted that the inclusion of WNT3A in prostate cancer cells augments Wnt signaling, and significantly increases self-renewal and sphere formation, which is correlated with β-catenin, CD44, and CD133 expression (37). Glycogen synthase kinase 3 beta (GSK3β) directly inhibits Wnt signaling *via* inducing β-catenin degradation. As a result, the Wnt pathway could be activated by the addition of AR79, a GSK3β inhibitor, which could lead to an increase in levels of ALDH and CD133 enrichment in a population of PCSCs *via* increasing nuclear β-catenin accumulation (38).

A recent study found that ASPM (an abnormal spindle-like microcephaly-associated protein) is a novel Wnt co-activator, which maintains cancer stem cells, such as the phenotype in prostate cancer by exerting Wnt signaling. ASPM binds to disheveled segment polarity protein 3 (DVL-3), a key upstream regulator of canonical Wnt signaling, and prevents it from being degraded by the proteasome, thereby strengthening protein stability and enhancing Wnt-induced β-catenin transcriptional activity in prostate cancer cells (39). Li et al. found that the expression of the period circadian regulator 3 (*PER3*) gene was negatively correlated with PCSC stemness *via* the targeting of the Wnt/βcatenin pathway. Therefore, low levels of PER3 promote brain and muscle ARNT-like 1 (BMAL1) expression, which in turn prompts enhanced β-catenin phosphorylation and activates the WNT/β-catenin pathway, thereby expanding the PCSCs (40). In addition, a CCL5 chemokine derived from tumor associated macrophages such as CCL5, not only support the rejuvenation and tumorigenicity of PCSCs, but also promote PCSC metastasis *via* activating the β-catenin/STAT3 signaling pathways (41). Knockdown of PTEN by shRNAs in prostate cancer cells leads to the activation of the PI3K/AKT pathway, which in turn increases the viability of cancer stem-like cell populations in prostate cancer (42). Hou et al.’s experimental data showed that up-regulation of thrombospondins 4 (THBS4) could promote the revitalization, proliferation, and tumorigenicity of prostate cells *via* activating the PI3K/AKT pathway (43). In addition, AKT can directly activate the Wnt pathway, not only promoting β-catenin transcriptional activity *via* phosphorylation of β-catenin at Ser552, but also inhibiting GSK3β function (44, 45). These findings suggest that Wnt/β-catenin and PI3K/AKT signaling work together to regulate PCSC stemness properties and targeting the pathways demonstrated potential therapeutic ways to eradicate prostate cancer.

Furthermore, the SHH pathway is associated with PCSC stemness regulation. Components of the SHH pathway, including GLI1 (GLI family zinc finger 1), PTCH1 (protein patched homolog 1), and SHH, are highly up-regulated in human prostate cancer tissues, compared with prostatic epithelium. SHH signaling is stimulated by androgen deprivation (46). The SHH signaling pathway promotes androgen-independent prostate cancer and causes therapy resistance *via* increasing ABC transporter levels (47). Inhibiting the SHH signaling pathways with anti-SHH antibodies diminishes prostate cancer growth (48). Chang et al.’s *in vivo* study demonstrated that overexpression of hedgehog signaling leads to the promotion of PCSC stemness properties (49). In contrast, the Notch signaling pathway also contributed to sustaining the stemness of PCSCs. CD54^+^ prostate cancer cells exhibited greater tumorigenicity, orb-forming capability, and resistance to the chemotherapeutic agent than CD54^–^ prostate cancer cells through activating the p38-Notch1 signaling pathway (35). Cheng et al. reported that cell–cell interaction governs PCSC stemness properties; mechanistically, the direct contact of prostate cancer cells with mesenchymal stem cells promotes sphere formation and tumorigenesis *via* activation of the Jagged1/Notch1 pathway both *in vitro* and *in vivo* (50).

Various epigenetic mechanisms, such as ubiquitination and modification of histone domains by methylation and acetylation, help to ameliorate the stem-like state in prostate cancer cells. For instance, the coregulation of EZH2 and breast cancer 1 (BRCA1) pathways is involved in the regulation of the stemness phenotype of PCSCs. Loss of EZH2 can induce down-regulation of BRAC1 that in turn triggers reprogramming capability, resulting in significantly increased tumor sphere formation, Levels of ALDH, and express stem cells phenotype in prostate cancer cells (51). Wu et al. demonstrated that when lymph node carcinoma of the prostate (LNCaP) cells are exposed to phenethyl isothiocyanate, the epigenetic modulator can alter the epigenome by decreasing the levels of H3K9ac by activating the PI3K/AKT pathway. These epigenetic and signaling mechanisms endow a cancer stem-like phenotype to LNCaP cells (52). Recent research has found that the bromodomain adjacent to zinc finger domain 2A (BAZ2A) is required for prostate cancer cells to acquire features similar to stem cells. The BAZ2A genomic population in prostate cancer cells correlates with the H3K14ac chromatin region; this BAZ2A/H3K14ac association is facilitated by BAZ2A-bromodomain (BAZ2A-BRD) *via* binding with H3K14ac. BAZ2A-BRD inhibits treatment, resulting in the prevention of the BAZ2A-BRD/H3K14ac interaction, which in turn hampers PCSC progression (53).

Cullin 4B (CUL4B), a member of the ubiquitin E3 ligase family, acts as tumor oncogene *via* inhibiting the tumor suppressor. However, the role of CUL4B in prostate cancer remains unclear. Jiao et al. found that activation of CUL4B in prostate cancer cells stimulates the stem-like state by up-regulating BMI1 *via* suppressing the miR200b/c expression. *In vitro* study revealed that activation of CUL4B in PC-3 and DU145 cells results in increased sphere formation and colony formation, and promotes pluripotent marker expression (54). In addition, *in vitro* study revealed that overexpression of basic transcription factor 3 (BTF3) could up-regulate BMI1 expression, which is a crucial mechanism for the acquisition of a stem cells-like phenotype in prostate cancer (55). Moreover, ubiquitin C-terminal hydrolase L1 (UCH-L1) and ubiquitin C-terminal hydrolase L3 (UCH-L3) differentially govern the stem-like traits in prostate cancer cells through regulating the PI3K/AKT pathway. Mechanistically, UCH-L1 and UCH-L3 inhibit the phosphorylation of downstream targets of the PI3K/AKT signaling pathway in DU145 cells, and up-regulate UCH-L1 or down-regulate UCH-L3 expression, which can in turn promote sphere formation ability, chemoresistance, and stem cell-related gene expression (56).

Androgen receptor (AR) signaling plays a vital role in prostate epithelium differentiation and development. Although androgen deprivation therapy (ADT) is the most often used treatment for prostate cancer, the majority of individuals eventually develop castration-resistant prostate cancer (CRPC), as the tumor is refractory to ADT. The primary CRPC is made up of both AR+ and AR– cell populations; however, AR^+^ cell populations are abundantly present in CRPC (57). In addition, ARs are highly expressed in differentiated prostate cancer (58). PCSCs mostly contain AR^–^ cell populations, as they are in an undifferentiated state (59). Sánchez et al. reported that androgen deprivation could induce the reprogramming of prostate cancer cells into stem-like cells. *In vitro* study demonstrated that androgen depletion in LNCaP cells could decrease the expression of AMP-activated protein kinase (AMPK) and increase stem cell marker expression including CD133, ABCB1A (ATP-binding cassette subfamily B member 1), and ALDH1A1, as well as activating stem cell-related transcription factors such as Nanog and OCT4. Therefore low levels of expression of ARs could activate stemness-related transcription factors, thereby increasing stemness features of PCSCs through an AMPK-dependent pathway (60). When prostate cancer is subjected to ADT, AR^–^ cell populations and PCSCs are not completely eradicated, and instead undergo some genetic alterations to possess stemness properties of CRPC (61). Yang et al. studied the relationship between AR and annexin A1 in prostate cancer cell lines. *In vitro* study results revealed that hampering the AR signaling ameliorates the migration of prostate cancer cells *via* the up-regulation of annexin A1 expression (62). Consequently, targeting AR alone might not provide therapeutic efficacy; hence, co-targeting the PCSCs with AR could improve the therapeutic efficacy against the prostate cancer.

## 4 Role of PCSCs in the development of anti-androgen resistance

The prostate gland consists of two layers of epithelial cells: luminal and basal cells. These two cell types exhibit different properties; the bulk of luminal epithelial cells highly express ARs and enable androgen signaling for cell survival, whereas basal cells are AR negative (63). Many studies have highlighted that overexpression of AR signaling relies on prostate cancer initiation and development (64, 65). Therefore, initially targeting the AR with an anti-androgen drug is efficacious; however, after ADT, most patients develop CRPC (66). Enzalutamide, a second-generation AR antagonist, has been shown in phase III clinical studies to prolong the survival of CRPC patients (67). However, even those who respond to enzalutamide at first eventually acquire chemoresistance and their disease advances (68). Therefore, identifying the underlying chemoresistance mechanism is imperative. Kregel et al. found that on AR-mediated repression, the pluripotent transcription factor SOX2 was significantly up-regulated. In this case, most of the tumors become resistant to the anti-androgen drug by evading targeted therapies through switching their lineage plasticity-like stemness state (69). In addition, Verma et al. also confirmed that androgen deprivation could induce the reprogramming of prostate cancer cells into stem-like cells. Treating the LNCaP cells with enzalutamide over the long term, exerting floating sphere formation, not only promotes the expression of stem cell markers, such as CD133 and ALDH1A1, but also modulates the transcriptional signature in prostate cancer cells for the acquisition of the stem cell phenotype by up-regulating Nanog and OCT4 (70). Mu et al. have reported that prostate CRPC cells develop resistance to enzalutamide by switching their phenotype lineage from AR-dependent luminal cells to AR-independent basal cells. This lineage plasticity switch is driven by the loss of tumor suppressor gene function, such as RB1 and TP53, is prompted by an increase in the expression of SOX2. It can be reversed by inhibiting SOX2 expression, thereby restoring the RB1 (RB transcriptional corepressor 1) and TP53 (tumor protein P53) function and inducing sensitivity lineage for anti-androgen drugs (71). The aforementioned Wnt/β-catenin pathway is involved in the regulation of stemness characteristics in PCSCs (72). Zhang et al. found that β-catenin is highly up-regulated in enzalutamide-resistant prostate cancer cells, and on activation of the Wnt/β-catenin pathway, stem cells markers are significantly increased. Furthermore, combination treatment with β-catenin inhibitor ICG001 and enzalutamide in a patient-derived LuCaP35CR xenograft model significantly reduced cancer cell proliferation, stem cell markers, and tumor growth compared to enzalutamide alone (73). Therefore, inhibiting PCSC stemness-associated signaling pathways and transcription factors circumvents anti-androgen drug resistance in prostate cancer. Subsequently, in the following sections we describe advanced therapeutic strategies for targeting PCSCs to ameliorate anti-androgen drug therapeutic function.

## 5 miRNAs

**Figure 2:**
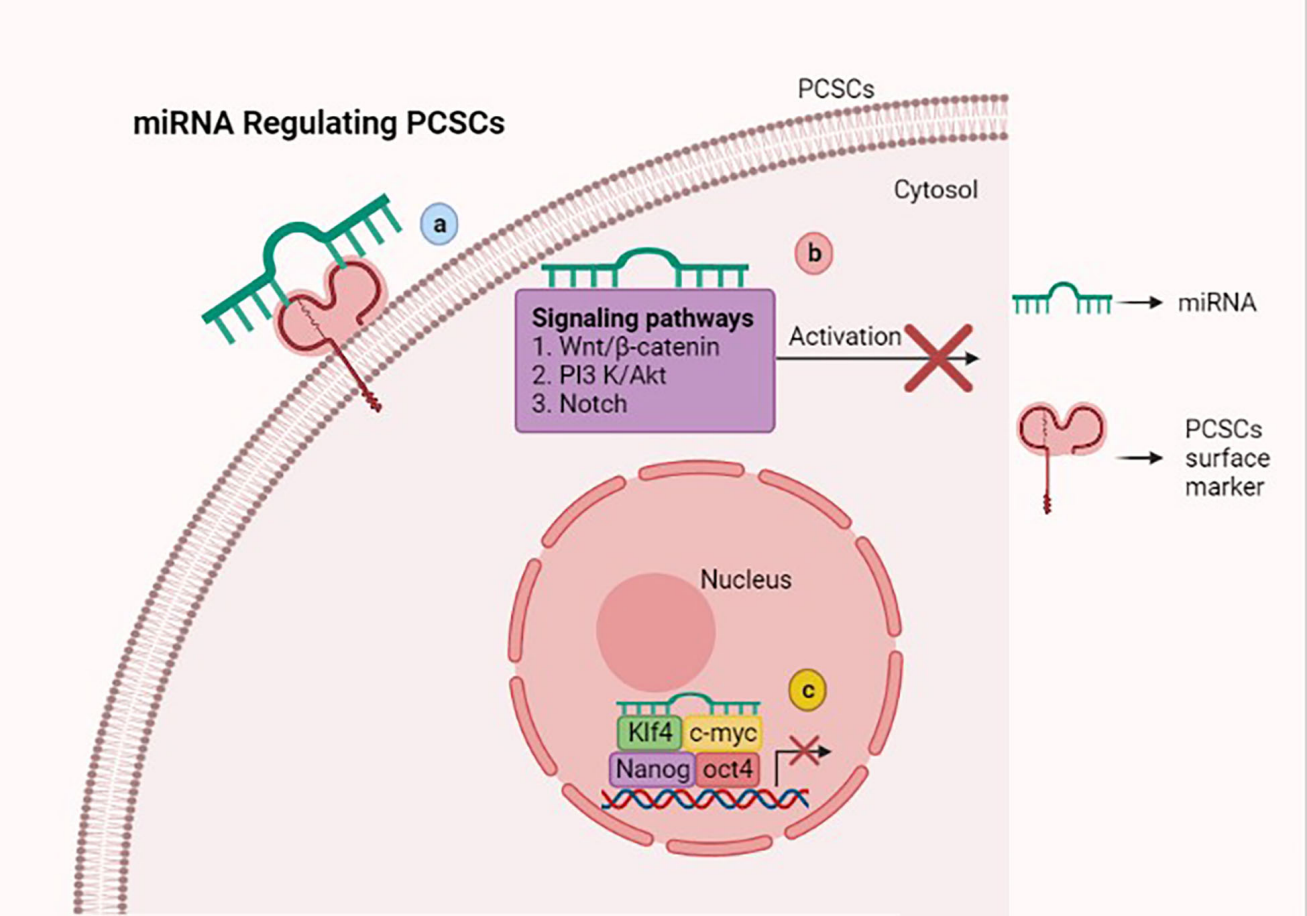
Scheme showing that microRNAs (miRNAs) regulate stemness features of prostate cancer stem cells (PCSCs) *via* various mechanisms. **(A)** miRNAs inhibiting stem-like traits of PCSCs *via* targeting the surface marker. **(B)** Attenuating Wnt/β-catenin, PI3K/AKT, and Notch signaling pathways with miRNAs suppresses PCSC growth and induces cell death. **(C)** miRNAs suppress the stem cell-related transcription factors such as OCT4, KLF4, Nanog, and *c-myc*, which hamper PCSC stemness properties. This figure was created with biorender.com.

MicroRNAs (miRNAs) are a small group of non-coding RNAs of 19–24 nucleotides, which are capable of regulating the tumorigenesis-related genes *via* binding the 3’ untranslated regions (UTRs) of the target gene mRNAs (74). Several studies suggest that miRNAs have the ability to manipulate the stemness properties of PCSCs *via* targeting surface markers, stem cell-related transcription factors, and signaling pathways (**Figure 2**).

CD44, a cell adhesion molecule, has been identified as a major cell surface marker in PCSs that expresses cancer stem cell-like properties, including tumorigenic, clonogenic, and metastatic properties (13). Liu et al. reported that miR-34a is a key negative regulator of CD44 in PCSs, and acts as a promising therapeutic agent against prostate cancer by repressing CD44, and, thus, inhibiting the metastasis of PCSs (75). Similarly, miR-708 negatively correlates with the CD44 subpopulation in PCSs and inhibits *in vitro* tumorigenicity by down-regulating CD44 expression (76). Further *in vitro* research reported that miR-143 and miR-145 hamper the tumor sphere formation by inhibiting stemness factors such as CD133 and CD44 in PC-3 PCSs (77).

The miRNA let-7c is down-regulated in prostate cancer and restoration of let-7c induces robust suppression of prostate cancer growth *in vitro* and *in vivo* (78). In a comprehensive study, Kong et al. demonstrated that overexpression of let-7c inhibits stemness of PCSs by suppressing EZH2, which plays a major role in cancer cell acquisition of stem cells’ signature. Indeed, after BR-DIM (metabolite 3,3′–diindolylmethane) treatment of prostate cancer cells, let-7c is up-regulated and EZH2 is down-regulated, which hampers the self-renewal and clonogenic properties of prostate cancer cells (79, 80). Jin et al. showed that the overexpression of miR-128 has negative effects on prostate cancer cells by hindering their proliferation, invasion, and sphere- and clonogenic-forming capacities. This can be achieved by targeting the cohort of stem cell regulatory factors including BMI1, Nanog, epidermal growth factor receptor (EGFR), and transforming growth factor beta receptor 1 (TGFBR1), which are all involved in maintaining the stemness of PCSs (81). Similarly, miR-100 has a negative impact on prostate cancer cells by down-regulating the oncogene argonaute 2 (AGO2), which can mediate stemness factors of cancer cells such as OCT4, Krüppel-like factor 4 (KLF4), and *c-myc*. The suppression of argonaute 2 with miR-100 expression inhibits the migration, invasion, and stemness of cancer cells (82). As previously mentioned, miR-143 and miR-145 attenuate tumorigenesis of PC-3 PCSs not only by targeting surface makers, but also by down-regulating stemness factors such as OCT4, *c-myc*, and KLF4 (77). In addition, miR-34a attenuates prostate cancer aggressiveness *via* down-regulating AR and Notch-1, which are highly expressed in prostate cancer (83). Guan et al. conducted an *in vitro* study based on miR-218 with prostate cancer cells, which revealed that miR-218 serves as a potential therapeutic target for prostate cancer by suppressing the expression of the GLI1 protein. This *in vitro* study elucidates the role of the GLI1 protein in the mechanism of tumor suppression of miR-218 in prostate cancer (84).

Several scientific reports have documented the role of miRNA in regulating prostate cancer stem cell-like characteristics by targeting various signaling pathways. Chang et al. reported that miR-7 acts as a critical inhibitor for prostate cancer. Mechanistically, a lower level of miR-7 expression existed in prostate cancer cells than in non-tumorigenic prostate epithelial cells, resulting in an over-expression of miR-7, which suppressed the stemness and tumorigenesis properties of PCSCs by targeting the KLF4/PI3K/AKT/p21 pathways (85). Similarly, miR-199a-3p exhibited tumor-suppressive functions in PCSs through the targeting of mitogen signaling-related biomolecules, including EGFR, *c-myc*, and cyclin D (86). Another *in vitro* study showed that miR-141 exerts the ability to inhibit tumor growth and metastasis properties of PCSs *via* targeting the Rho GTPase signaling-associated components, including CDC42, CDC42EP3, RAC1 (ras-related C3 botulinum toxin substrate 1), and ARPC5 (actin-related protein 2/3 complex subunit**)** (87). Furthermore, the expression of miR-320 in prostate cancer cells significantly inhibits stem cell-like characteristics by down-regulating the Wnt/β-catenin signaling pathways *in vitro* and *in vivo* (88).

Numerous studies reported that miR-200 families could regulate the EMT phenotype in PCSCs. Emerging evidence suggests that overexpression of platelet-derived growth factor-D (PDGFD) in PSCSs could mediate acquisition of the EMT phenotype. In PC-3 cells, miR-200 was significantly down-regulated and the expression of ZEB1, ZEB2, and SNAIL2 was up-regulated when exposed to PDGFD. As a result, reconstruction of miR-200 in PC-3 + PDGFD cells inhibits the EMT phenotype by suppressing expression of ZEB1, ZEB2, and snail2 (89). Simultaneously, SLUG is a key regulator of EMT initiation and mesenchymal differentiation, inducing the expression of miR-1 and miR-200 in PC-3 cells, thus suppressing the EMT *via* SLUG-dependent mechanisms and inhibiting tumorigenesis *via* SLUG-independent mechanisms (90). Furthermore, cancer-associated fibroblasts (CAFs) are a strong determinant for eliciting a redox-dependent EMT phenotype in prostate cancer cells. Ectopic expression of miR-205 prevents CAF-mediated EMT, thus hampering cell invasion, stemness characteristics, and tumorigenicity *in vitro* and *in vivo* (91).

Alternatively, miRNAs expression is directly proportional to PCS growth. Fan et al. reported that up-regulation of miR-143 expression in PCSs ameliorates the stemness properties of prostate cancer cells, such as differentiation and metastasis, by suppressing FNDC3B expression, which regulates cell motility (92). At the same time, ectopic expression of miR-1301–3p enhances expansion of PCSs by targeting the GSK3β and SFRP1 pathways and increasing the expression of stemness-associated factors, such as OCT4, KLF4, Nanog, SOX2, and *c-myc* (93). Further *in vitro* studies confirmed that down-regulation of miR-601 in PCSCs counteracts proliferation, migration, and invasion *via* promoting KRT5 and subsequently blocking the Wnt pathway (94).

NUMB is an evolutionarily conserved protein, widely present in mammalian tissues, and it plays a crucial role in cell fate determination, proliferation, differentiation, and migration (95). In addition, NUMB exerts a tumor suppressor function and inhibits the stemness of PCSCs (96). MiR-543 and miR-9–5P potentially target the NUMB and attenuate NUMB expression in PC-3 cells, promoting stemness and metastasis of PCSCs (97, 98). The role miR-424 plays depends on the cancer. In ovarian cancer, miR-424 can act as a tumor suppressor by inhibiting cell migration, invasion, and EMT *via* down-regulating the expression of doublecortin-like kinase 1 (DCLK1) and MYB (99, 100). Similarly, in breast cancer, miR-424 suppresses the cell invasion and stem cell traits under hyperglycemic conditions through the attenuated inhibitory function of miR-424 on CDC42, leading to the activation of PR/SET domain 14 (PRDM14), which is a repressor of pluripotent transcription factors (101, 102). However, miR-424 exhibits oncogenic effects in prostate cancer. Dallavalle et al. reported that miR-424 expression was up-regulated in prostate tumors, promoting EMT and stem-like features by silencing COP1, which mediates the STAT3 accumulation to activate stemness factors (103). In addition, circulating extracellular vesicles release higher levels of miR-424 in metastatic prostate cancer that drive the acquisition of stemness features and tumorigenesis properties in prostate cancer (104). A recent study found that miR-375 is significantly elevated in CRPC patients compared with normal prostate cancer patients. Overexpression of miR-375 in DU145 and PC-3 cells promotes cell proliferation, migration, invasion, and enzalutamide drug resistance, and inhibits cell apoptosis *via* targeting the phosphatase non-receptor type 4 (PTPN4), resulting in the activation of STAT3. Targeting miR-375 with e-375i could inhibit prostate cancer cell proliferation, migration, and invasion, and promote cell apoptosis and enzalutamide drug sensitivity in prostate cancer *via* down-regulating STAT3 expression by up-regulating PTPN4 expression (105). As a result, down-regulating miR-143, miR-1301-3p, miR-601, miR-543, miR-9-5P, miR-424, and miR-375 in PCSCs could be a potential therapeutic method for treating prostate cancer.

## 6 Support of nanotechnology

**Figure 3:**
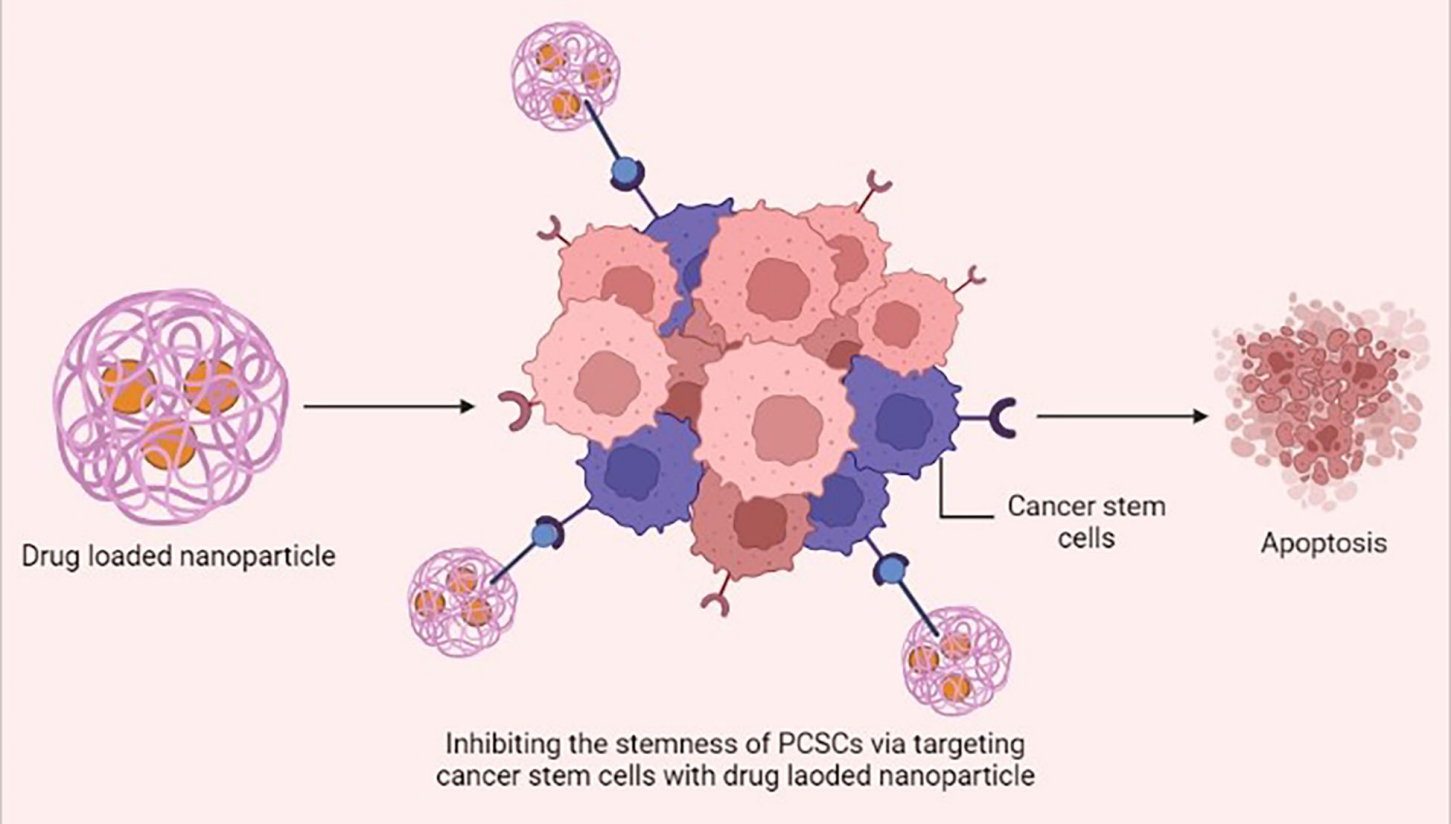
Drug-loaded nanoparticles inhibiting the stemness of prostate cancer stem cells (PCSCs) *via* specifically targeting the cancer stem cells and inducing apoptosis. This figure was created with biorender.com.

Nanotechnology has become a rapidly growing field in recent years, with potential applications in academic and industrial contexts for the purpose of developing novel materials at the nanoscale (106). The unique size, shape, and desirable surface modification of nanomaterials are considered to have potential for therapeutic nanomedicine and could be extensively used as a nano drug carrier for prostate cancer therapy (107). The clinical utility of biochemical inhibitors is hampered by their hydrophobicity and poor pharmacokinetics; in order to increase their bioavailability, these inhibitors are conjugated with nanoparticles and they attenuate stemness of PCSCs (108) (**Figure 3**). *Cis-*dichlorodiamminoplatinum (CDDP) is a potential anti-tumor agent for various cancers. Malek et al. fabricated CDDP-loaded hyaluronic acid (HA) nanoparticles, as a substrate for CD44, by using a drug-induced ionic gelation technique. *In vitro* study results revealed that HA-CDDP could act as a targeted drug delivery system and significantly prevent the sphere- and colony-forming capacity in PC-3 and DU145 cell lines (109).

Zhou et al. evaluated *N*-(2-hydroxypropyl)methacrylamide (HPMA) copolymer-based combination therapy with the PI3K/mTOR inhibitor GDC-0980 and the traditional chemotherapy agent docetaxel, both *in vitro* and *in vivo*. *In vitro* study indicated that HPMA + GDC-0980 could inhibit sphere-forming capacity and decrease the CD133 PCSC-rich population in the PC-3 cell line. In the PC-3 tumor xenograft mice model, combination treatment of HPMA + GDC-0980 with docetaxel significantly prolonged mice survival compared with treatment without docetaxel administration (110). As such, HPMA + cyclopamine treatment could also reduce sphere-forming capacity and the percentage of CD133 PCSCs in PC-3 and RC-92a/hTERT cells. *In vivo* study highlighted that combination administration of HPMA + cyclopamine with docetaxel led to significantly decreased tumor volume compared with the single drug in PC-3 tumor xenograft mice models (111). Yang et al. combined the paclitaxel and cyclopamine with poly(ethylene glycol)-*block*-poly(2-methyl-2-carboxyl-propylene carbonate) (mPEG-b-PCC) nanoparticles. Both the drug-loaded nanoparticles exhibited sustained drug release and suppressed the colony-forming ability in PC-3 cells by up-regulating tumor suppressor miRNAs. The combination of paclitaxel and cyclopamine-loaded nanoparticles induced significant tumor inhibition in PC-3 tumor xenograft mice models compared with monotherapy (112). All these findings suggest that combination therapy targeting PCSCs is a promising approach to enhancing therapeutic effect against prostate cancer.

Interestingly, similar to drug-loaded nanomaterials, some nanomaterials themselves can have an antitumor effect. Recent research found that graphene oxide (GO) potentially inhibits the sphere-like capacity of not only PCSCs, but also ovarian cancer cells, glioblastoma cells, lung cancer cells, and pancreatic cancer cells. Notably, GO is non-toxic for normal fibroblasts (113). Cuprous oxide (CO) nanoparticles naturally exhibit a potential antitumor function as a chemotherapeutic agent (114). Wang et al. reported that CO nanoparticles could selectively induce cell death and inhibit prostate cancer cell proliferation both *in vitro* and *in vivo* without damaging normal prostate epithelial cells. Correspondingly, CO nanoparticles can also prevent stemness of PCSCs *via* inhibiting the Wnt signaling pathway (115).

## 7 Chemotherapy

Chemotherapy is a conventional treatment in cancer therapy involving administration of chemotherapeutic agents to eradicate tumor cells. Several scientific reports have demonstrated that these agents can act as a potential inhibitor for various endogenous signaling pathways that are involved in regulating PCSCs stemness phenotype, thereby possessing potential therapeutic value for prostate cancer treatment. A recent study found that the JAK/STAT signaling pathway was up-regulated in CRPC patients and conferred resistance to AR-targeted therapies. Inhibiting the JAK/STAT pathway with a pharmacological agent resensitizes the AR-resistant prostate tumor to anti-androgen drugs (116). In addition, ectopic activation of the JAK/STAT pathway in LNCaP cells enables transition to a stem-like state and thereby induces the resistance to enzalutamide drugs. Inhibiting the JAK/STAT pathway restores luminal lineage and sensitizes the prostate tumor to enzalutamide drugs (117). Therefore, the JAK/STAT pathway plays an important role in regulating PCSC phenotype. Canesin et al. showed that direct inhibition of the STAT3 transcription factor with galiellalactone in a DU145 cell line could reduce the expression of stem cell markers and sphere and colony formation, and also decrease the viability of the docetaxel-resistant cell line (118). An *in vitro* study demonstrated that administration of capsaicin to PC-3 and DU145 cells could down-regulate PCSCs markers, such as CD44, CD133, ALDH1A1, SOX2, OCT4, and Nanog, and suppress cell growth *via* inhibiting the Wnt/β-catenin pathways (119). In addition, saikosaponin D could act as a potential chemotherapeutic agent for CRPC through the suppression of stemness abilities, such as self-renewal, sphere formation, and EMT, by blocking the Wnt/β-catenin signaling pathways by down-regulating GSK3b phosphorylation (120). The combination therapy with apigenin and cisplatin stimulates apoptosis and anti-migration of CD44 PCSCs. This combination therapy promotes apoptosis by up-regulation of apoptotic factors p53, p21, and apoptotic protease activating factor 1 (APAF1); down-regulation of anti-apoptotic factor BCL-2; and suppression of the PI3K/AKT pathway (121). Wang et al.’s *in vitro* study demonstrated that exposure of shikonin to PC-3 and DU145 cells could inhibit proliferation, migration, and invasion, and induce cell death through a mitochondrial-mediated apoptosis pathway. Furthermore, shikonin could reverse the resistant state of cabazitaxel by inhibiting ABCG2 and ALDH3A1 expression, which are linked with drug resistance. Therefore, the combination of shikonin and cabazitaxel causes a synergistic effect and can enable the use of lower doses of cabazitaxel for CRPC therapy (122). Comparably, Liu et al. found that inhibition of the Notch signaling pathway could improve chemosensitivity in PCSCs *via* decreasing the ABCC1 expression (123). According to this study, Wang et al. examined the effect of docetaxel on a PC-3 cell line with γ-secretase inhibitor (GSI), a potent inhibitor of the Notch pathway. *In vitro* study results revealed that the combination of docetaxel and GSI significantly inhibited cell growth and sphere formation, and induced apoptosis and cell cycle arrest in PCSCs when compare with docetaxel alone (124). Furthermore, PCSC growth could be attenuated by rottlerin treatment, which can activate the autophagy mechanism *via* up-regulation of the AMPK pathway and induction of apoptosis *via* inhibiting the PI3K/AKT/mTOR pathway (125). *In vitro* research showed that neferine inhibits the cell viability and migration of CD44 PCSCs *via* mediating JNK and p38 MAPK phosphorylation, and blocking PI3K and NF-ĸβ signaling (126). Bahmad et al. developed and examined the antitumor mechanism of synthetic retinoid ST1926 on PCSCs. Results indicated that ST1926 reduced cell proliferation, migration, and invasion and also mediated p53‐independent apoptosis *via* early DNA damage in PCSCs (127). Details of current clinical trials utilizing monotherapy and combinational therapy have been highlighted in **Tables 1**, **2**.

**Table 1:** Current clinical trials on monotherapies.

Study no.	Clinical trial identifier number	Therapy name	Phase information	Study purpose	Clinical trial outcome	Reference
1	NCT02392611	BET inhibitor GS-5829 monotherapy	Phase IB study	GS-5829, an oral bromodomain and extra terminal inhibitor, was studied both by itself and in tandem with enzalutamide in mCRPC Its effectiveness and safety were assessed	In individuals with mCRPC, GS-5829 was usually well tolerated but showed limited efficacy and no dose-proportional changes in plasma concentrations	(128)
2	NCT02991911	MEDI3726, a prostate-specific membrane antigen-targeted antibody–drug conjugate	Phase I study	This study assessed MEDI3726 monotherapy in patients who had mCRPC after disease progression on abiraterone and/or enzalutamide and taxane-based chemotherapy	Modest clinical activity was observed, particularly at the higher dosages tested, in a patient population with poor results in general; the treatment’s duration was curtailed and further planned dose escalation was hindered by emergent treatment-related toxicities	(129)
3	NCT02799745	Enzalutamide monotherapy	A phase II, open-label, randomized clinical trial	The aim of this study is to evaluate the effectiveness and reliability of enzalutamide monotherapy plus AS vs. AS in individuals with low-risk or medium-risk prostate cancer	As per the outcomes of this randomized clinical study, patients with low- or intermediate-risk localized prostate cancer responded significantly to enzalutamide monotherapy and tolerated it well. For patients undergoing AS, enzalutamide monotherapy may provide an alternative therapy approach	(130)
4	NCT02319837	Enzalutamide plus leuprolide and enzalutamide monotherapy in high-risk non-metastatic hormone-sensitive prostate cancer with rising PSA levels after local therapy	A phase III randomized study	The trial’s primary goal is to compare the effectiveness of enzalutamide plus leuprolide (LHRHa), enzalutamide monotherapy, and monotherapy LHRHa	Predicated on phase findings, enzalutamide monotherapy may have a therapeutic advantage over LHRHa as it induces a robust and long-lasting PSA response	(131)
5	NCT02787005	Pembrolizumab for treatment-refractory metastatic castration-resistant prostate cancer	Multicohort, open-label phase II KEYNOTE-199 study	PD-L1-positive mCRPC has previously been treated with pembrolizumab and proven to exhibit anti-tumor efficacy (mCRPC). In this study, three parallel cohorts of a larger mCRPC population were used to evaluate the anti-cancer efficacy and safety of pembrolizumab monotherapy	A minority of patients with RECIST-measurable and bone-predominant mCRPC who had previously received treatment with docetaxel and targeted endocrine therapy respond to pembrolizumab monotherapy with anti-tumor efficacy and a tolerable safety profile. The OS estimates are optimistic, and the observed reactions appear to be durable	(132)
6	NCT02390063	ChAdOx1-MVA 5T4 vaccine	Phase I clinical trial	To determine the safety and immunogenicity of novel 5T4 viral vectored vaccination regimens in early-stage prostate cancer	The ChAdOx1-MVA 5T4 vaccine is being evaluated in feasibility trials owing to its excellent clinical efficacy and T-cell responses that were both observed in the prostate gland and the blood.	(133)
7	NCT03148795	Talazoparib monotherapy	An open-label, phase II trial	The PARP inhibitor talazoparib was examined in this study in mCRPC with DDR-HRR mutations	In men with advanced metastatic castration-resistant prostate tumors with DDR-HRR gene mutations who had a lot of pretreatments, talazoparib demonstrated persistent anti-tumor efficacy. The exploration of talazoparib in greater, randomized clinical studies, particularly in individuals with non-*BRCA* gene mutations, is supported by the drug’s favorable benefit–risk profile	(134)
8	NCT02740985	AZD4635 (an adenosine A2A receptor antagonist) as monotherapy or combined with durvalumab	Phase IA/B	This is the first in-human phase IA/B research investigating the oral A2AR antagonist AZD4635 both as a monotherapy and in combination with the anti-PD-L1 monoclonal antibody durvalumab for safety, tolerability, PK, and preliminary clinical efficacy	In all prostate cancer patients, AZD4635 was well tolerated as a monotherapy and in combination with durvalumab	(135)

**Table 2:** Current clinical trials on combination therapies.

Study no.	Clinical trial identifier number	Therapy name	Phase information	Study purpose	Clinical trial outcome	Reference
1	ISRCTN06890529	ADT combined with concurrent radiation therapy	Randomized clinical trial	To ascertain whether or not combining local EBRT with ADT for the local treatment of the primary prostatic tumor increases OS	OS was the main objective Time until PSA progression was the secondary goal Crude and adjusted analyses were used to gauge the effectiveness of the treatment	(136)
2	NCT00309985	Chemohormonal therapy in mHSPC	Randomized phase III E3805 CHAARTED trial	Docetaxel combined with ADT extends the lives of some patients with metastatic hormone-sensitive prostate cancer. Therefore, with more in-depth follow-up and an emphasis on tumor volume, we provide the findings from the CHAARTED trial	For patients with high-volume disease, the clinical effect of CHT in extending OS was verified; however, for patients with low-volume disease, no OS improvement was perceptible	(137)
3	NCT01946204	Combined ADT and apalutamide	Phase III trial	In this trial, the effectiveness of apalutamide in men with castration-resistant non-metastatic prostate cancer who were at high risk of metastasis is being assessed	Apalutamide (an androgen synthesis inhibitor) considerably outlived a placebo in terms of metastasis-free survival and time to symptomatic progression in males with non-metastatic castration-resistant prostate cancer	(138)
4	NCT02799602	Combination of ADT with darolutamide and docetaxel	Phase III trial	To assess whether or not patients with mHSPC cancer would have a higher chance of survival if they received docetaxel, ADT, and darolutamide together as a combination therapy	Compared with placebo plus ADT and docetaxel, overall survival was considerably longer with the combination of darolutamide, ADT, and docetaxel, and the inclusion of darolutamide improved important secondary end points	(139)
5	NCT00268476	Abiraterone acetate and prednisolone with or without enzalutamide for high-risk nmPC	Phase III trial	Two randomized controlled phase III trials provided fresh data that we analyzed A multiarm, multistage platform approach was used to evaluate the effectiveness of combining abiraterone and prednisolone with ADT in the nmPC patient population	In conclusion, ADT combined with combination therapy significantly improves metastases-free survival and OS for men with high-risk nmPC compared with ADT alone The new standard of care for nmPC with high-risk characteristics should be 2 years of abiraterone and prednisolone coupled with ADT and, if required, radiation	(140)
6	NCT02058706	Enzalutamide vs. bicalutamide combined with ADT	Randomized clinical trial, phase II trial	To assess, in men with mHSPC and with a subset analysis of black patients, the efficacy of enzalutamide vs. bicalutamide in conjunction with ADT	The main outcome was the PSA response rate at 7 months, which was previously recognized as a proxy for OS results Adverse effects, the amount of time until PSA progression, and OS were secondary end points	(141)
7	NCT02460224	LAG-3 inhibitor ieramilimab (LAG525) and anti-PD-1 spartalizumab (PDR001)	Phase II/III trial	Phase I’s main goal was to calculate the recommended phase II dosage (RP2D) or maximum tolerated dose (MTD) of ieramilimab, both as a solo drug and in combination with spartalizumab The characterization of the safety and tolerability of ieramilimab in combination with spartalizumab, the measurement of PK, and the evaluation of preclinical anti-cancer efficacy were important secondary goals	Ieramilimab, both alone and in combination with spartalizumab, was well tolerated Ieramilimab in combination with spartalizumab had a similar toxicity profile to the spartalizumab alone Combination therapy produced only weak antitumor effects	(142)
8	NCT02312557	Pembrolizumab with enzalutamide	Phase II trial	In a single-arm phase II research, evaluation of the combination of enzalutamide and the PD-1 inhibitor pembrolizumab in 28 men with metastatic castration-resistant prostate cancer was done	In mCRPC, pembrolizumab exhibits activity when used with enzalutamide No tumor PD-L1 expression or DNA repair deficiencies were required for the responses to be profound and long-lasting	(143)

## 8 Immunotherapy

Immunotherapy is an alternative potential therapeutic option, associated with immunological agents such as vaccines, antibodies, and immune cells, and chimeric antigen receptor T (CAR-T) cell therapy for the management of advanced cancer (144). The prostate stem cell antigen (PSCA) is a notable antigen; owing to its overexpression in prostate cancer, and exclusively in metastatic tissues; thus, it is a promising candidate for prostate cancer immunotherapy (145, 146). *In vivo* research showed that PSCA vaccination could mediate the expression of CD4 and CD8 T cells and induce a long-term protective immune responses against advanced prostate cancer (147). The stemness of PCSCs is regulated by angiogenin and plexin B2: the bioactive 3′-end fragments of 5S ribosomal RNA are synthesized in order to inhibit the function of angiogenin and plexin B2 with monoclonal antibodies, resulting in reduced stemness of PCSCs and PCSCs sensitized to chemotherapy (148). Wang et al. investigated an innovative immunotherapeutic platform based on non-peptide-stimulated dendritic cells-activated cytokine-induced killer (NP-DC-CIK) cells and immunogenic peptide-stimulated P-DC-CIK cells. These immunogenic peptides were derived from PCSC-related antigens such as CD44 and the epithelial cell adhesion molecule (EpCAM). *In vitro* and *in vivo* study results showed that peptide-stimulated dendritic cells-activated cytokine-induced killer (P-DC-CIK) cells exhibited significant cytotoxicity against PCSCs. Cytokine-induced killer cells are specifically activated on dendritic cell sensitization by immunogenic peptides with PCSCs, which significantly prompts *in vitro* cytotoxicity against PCSCs and induces an *in vivo* anti-tumor effect (149). Alternatively, Seki et al. reported that co-cultured KHYG-1 human NK cells can preferentially target PCSCs *via* the TRAIL/DR5 signaling pathway. *In vitro* data showed that KHYG-1 cells could induce more cytotoxicity against LNCaP stem-like cells than androgen-dependent LNCaP cells (normal prostate cancer cells) (150).

**Figure 4:**
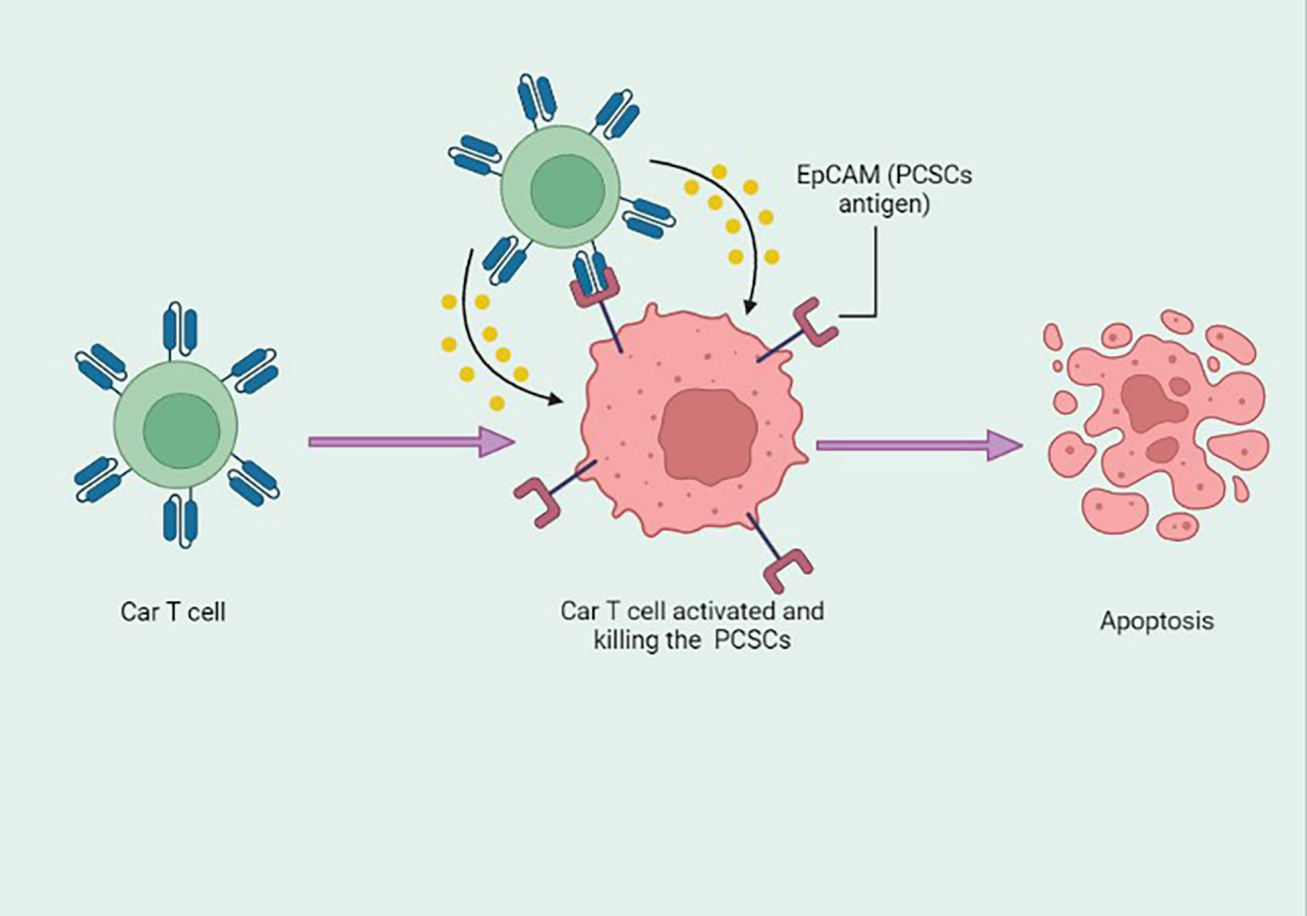
Epithelial cell adhesion molecule (EpCAM) antigen-based engineered CAR-T cells specifically targeting the prostate cancer stem cells (PCSCs) and inducing apoptosis. This figure was created with biorender.com.

Recently, CAR-T cell therapy has garnered significant research interest as a cancer therapy, owing to its profound target specificity and low major histocompatibility complex restriction in immunotherapy and its ability to exert cellular adoptive immunotherapy. With the advancement of genetic engineering technology, chimeric antigen receptors (CARs) can be designed according to the antigen specific to the target cell and introduced into patient T lymphocytes by *in vitro* transduction (151). To illustrate, Dang et al. designed a novel immunotherapy strategy for targeting the EpCAM, a PCSC antigen. It was designed in EpCAM-specific CARs and introduced into human peripheral blood lymphocytes (PBLs); results suggested that EpCAM-specific PBLs significantly inhibit PC-3 tumor cell growth *in vitro* and *in vivo* by targeting PCSCs (**Figure 4**) (152). PCSCs are resistant to radiation therapy, specifically fractionated irradiation, which significantly up-regulates the immunoregulatory protein B7-H3 on PCSCs compared with prostate cancer cells. In order to, Zhang et al. developed B7-H3 CAR-T cells that can potentially target radiation-resistant PCSCs. Hence, the combination treatment of B7-H3 CAR-T cells with radiation therapy could induce robust cytotoxicity against PCSCs *in vitro* and *in vivo* (153). Another study demonstrated that PSCA-specific engineered CAR-T cells in combination with interleukin-2 (IL2) eradicate PSCA-expressing cancer cells (154).

## 9 Concomitant immunotherapy strategy

PCSCs are responsible for tumor initiation, growth, and relapse in prostate cancer; consequently, TLR3 activation-based immunotherapy using polyinosinic:polycytidylic acid could be a good option for treatment, as it generates apoptosis and an inflammatory response in tumor cells (155).

**Figure 5:**
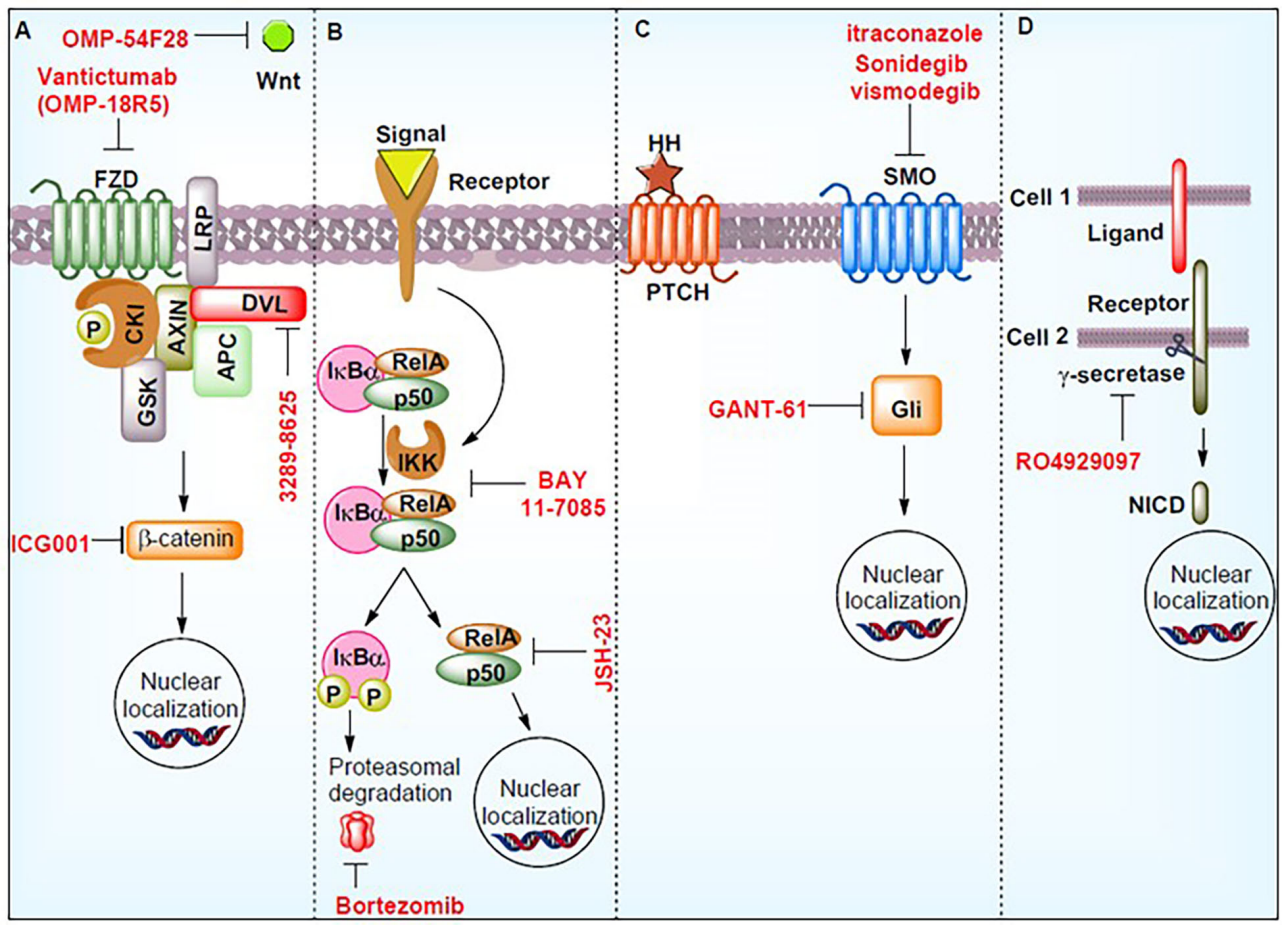
Signaling pathways controlling prostate cancer stem cells (PCSCs) and the work of inhibitors in suppressing these pathways. These molecules may act as potential therapeutics. The figure demonstrates the hedgehog signaling pathway (HH), notch intracellular domain (NICD), phosphorylation (P), smoothened (SMO), Wnt signaling pathways (Wnt), nuclear factor of kappa light polypeptide gene enhancer in B-cells inhibitor, alpha (IkBα), RELA Proto-Oncogene (RelA), Frizzled (FZD), casein kinase I (CKI), axin (AXIN), APC regulator of the WNT signaling Pathway (APC), disheveled (DVL), glycogen synthase kinase (GSK), and inhibitor of nuclear factor kappa B kinase (IKK). Reproduced with permission from (157).

The plasticity of cancer stem cells is responsible for tumor phenotype progression and response to therapy; therefore, an understanding of biomarkers is important. Understanding the molecular basis has led to the development of patient therapies (156). PCSCs play a central role in the development of treatment resistance and subsequent disease progression. PCSCs exhibit surface markers, such as ALDH, CD133, and CD44, possessing states of self-renewal, colonization and recurrence (157). Various other cellular incidents promote PCSCs. It is likely that signaling pathways, such as Wnt/β-catenin, hedgehog, NF-κB, and Notch, ABC transporters, and the tumor microenvironment could be the putative target(s) for PCSCs (158). **Figure 5** demonstrates the signaling routes controlling PCSCs and the work of inhibitors in suppressing these pathways (157). Therefore, targeting PCSCs could be an improved approach for the treatment of CRPC, and several inhibitors, antibodies, and other combinative therapy could be evaluated with clinical trials.

Studies have found these therapies to be effective in improving survival outcomes for prostate cancer patients, although full treatment is still challenging for clinicians owing to the development of drug-resistant features over time. However, understanding PCSCs’ differentiation, identification, and characterization, and their isolation through pathway-dependent expansion, is of great importance for therapeutic opportunities.

## 10 CRISPR/Cas9 system

**Figure 6:**
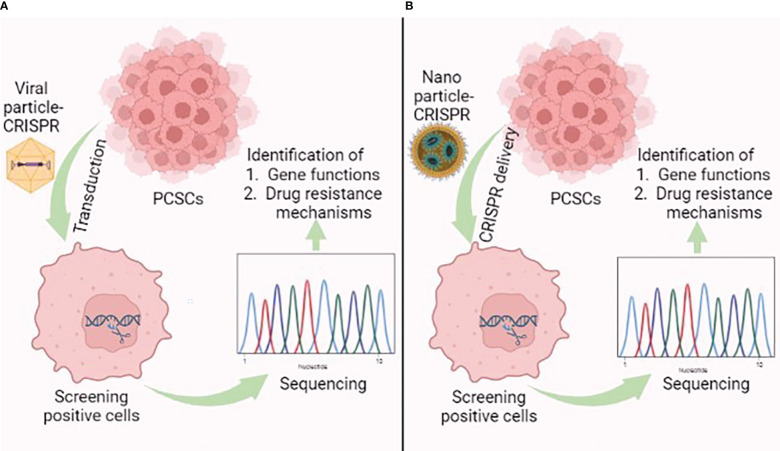
Graphical illustration showing the application of the **(A)** viral-mediated and **(B)** nanoparticle-mediated CRISPR/Cas9 delivery system in prostate cancer stem cells (PCSCs). This figure was created with biorender.com.

With its profound limited off-targets and high accuracy, the CRISPR/Cas9 system represents an unbiased genome editing system. The system has been widely exploited to elucidate molecular mechanisms of cancer progression *via* gene knockout or knockdown (159). Single guide RNA (sgRNA), and Cas9 are essential components of the CRISPR system, which have been extensively delivered by viral and non-viral approaches (160). In prostate cancer therapy, the viral- and non-viral-mediated CRISPR/Cas9 delivery system is a pioneering tool that performs systematic genome-wide genetic screening to identify the function of novel genes and drug resistance mechanisms, which in turn enables new drug development for feasible therapeutic approaches (**Figure 6**) (161). Fei et al. used a lentiviral vector to deliver CRISPR components to prostate cancer cells. The use of genome-wide CRISPR-knockout screens found that the heterogeneous nuclear ribonucleoprotein L (*HNRNPL*) gene is essential and is required for prostate cancer cell growth. The gene can directly regulate alternate splicing of its RNA targets, including the AR-encoding region (162). Das et al. performed genome-wide CRISPR-interference screens in metastatic prostate cancer models and found two prostate cancer-specific driver genes, *KIF4A*, and *WDR62*. Both genes induced the phenotypically aggressive prostate cancer in both *in vitro* and *in vivo* models (163). Lei et al. found that CDK12 is conservatively required for prostate cancer cell growth by performing a kinome-scale CRISPR/Cas9 screen. Repression of CDK12 with covalent inhibitor THZ531 exerts an anti-prostate cancer effect; therefore, CDK12 represents a druggable target of prostate cancer (164).

Alternatively, an *in vitro* study demonstrated that CRISPR/Cas9 system-mediated lipocalin 2 gene knockout decreased PC-3 cell proliferation and migration, as well as increasing the sensitivity of cisplatin drug-induced apoptosis (165). Rushworth et al. conducted an *in vivo* study based on a prostate cancer orthograft model sensitized with the genome-wide CRISPR/Cas9 knockout system under docetaxel treatment. Screening results highlighted that the combination of docetaxel with loss of TCEAL1 exhibited a higher therapeutic value than docetaxel alone. Therefore, silencing the TCEAL1 expression improves docetaxel efficacy in prostate cancer treatment (166). AR inhibitors such as apalutamide and enzalutamide are a mainstay of prostate cancer therapy. Palit et al. showed through the genome-wide CRISPR/Cas9 screen that on the loss of TLE3, prostate cancer cells are resistant to AR inhibitors. Notably, consistent with the interaction of TLE3 and AR at the glucocorticoid receptor (GR) locus, loss of TLE3 results in the up-regulation of GR expression, which in turn leads to resistant AR inhibitors. Thus, this study provides novel insights on GR-mediated AR inhibitor resistance, as regulated by TLE3 (167). Correspondingly, it was found through performing a kinome-scale CRISPR/Cas9 screen in CWR-R1 prostate cancer cells that an activated BRAF signaling pathway is resistant to enzalutamide. Inhibition of BRAF or downstream components of MAPK, such as MEK and ERK, ameliorate the enzalutamide sensitivity in prostate cancer cells. As a result, the combined inhibition of MAPK and AR pathways provides a potential therapeutic effect in BRAF-mutated prostate cancer patients (168).

In prostate cancer, NANOG is highly associated with cancer stem cell traits and resistance to androgen deprivation therapy. Kawamura et al. generated a Nanog and Nanogp8 knockout prostate cell line DUI45 using the CRISPR/Cas9 system. Nanog and Nanogp8 knockout significantly impaired the malignant potential, sphere formation, and drug resistance of cancer stem cells compared with the control DU145 cell line (169). However, all experiments mentioned above were conducted by the viral-mediated CRISPR/Cas9 delivery system, which comes with a risk of immunogenicity. As a result, non-viral particles are a promising vehicle for delivering CRISPR/Cas9 components. For example, Zhen et al. designed an aptamer–liposome–CRISPR/Cas9 chimera for specifically targeting prostate cancer. The chimera-inserted RNA aptamer acts as an antigen for prostate cancer, and the CRISPR/Cas9-loaded cationic liposomes are attached to the aptamer. The aptamer–liposome–CRISPR/Cas9 chimera significantly binds to LNCaP cells and attenuates cancer cell growth *via* silencing the polo-like kinase 1, which is a cell survival gene (170). Hence, targeting stemness-related transcription factors or signaling pathways with a non-viral-based CRISPR/Cas9 system could be a potential therapeutic strategy for prostate cancer treatment.

## 11 Photothermal ablation therapy

In recent years, photothermal ablation (PTA) therapy has become a promising treatment in the management of various types of cancer with minimal invasiveness. In this approach, light energy is converted into heat energy by exploiting photothermal agents with a high-power near-infrared (NIR) laser to place cancer cells in remission (hyperthermia) (171). Cancer cells are more sensitive than normal cells to heat stress owing to the different pattern expressions of heat shock proteins that are involved in the cellular defense mechanism. As a result of elevating the tumor microenvironment temperature, cancer cells are irretrievably damaged because of the denaturation of proteins (172).

**Figure 7:**
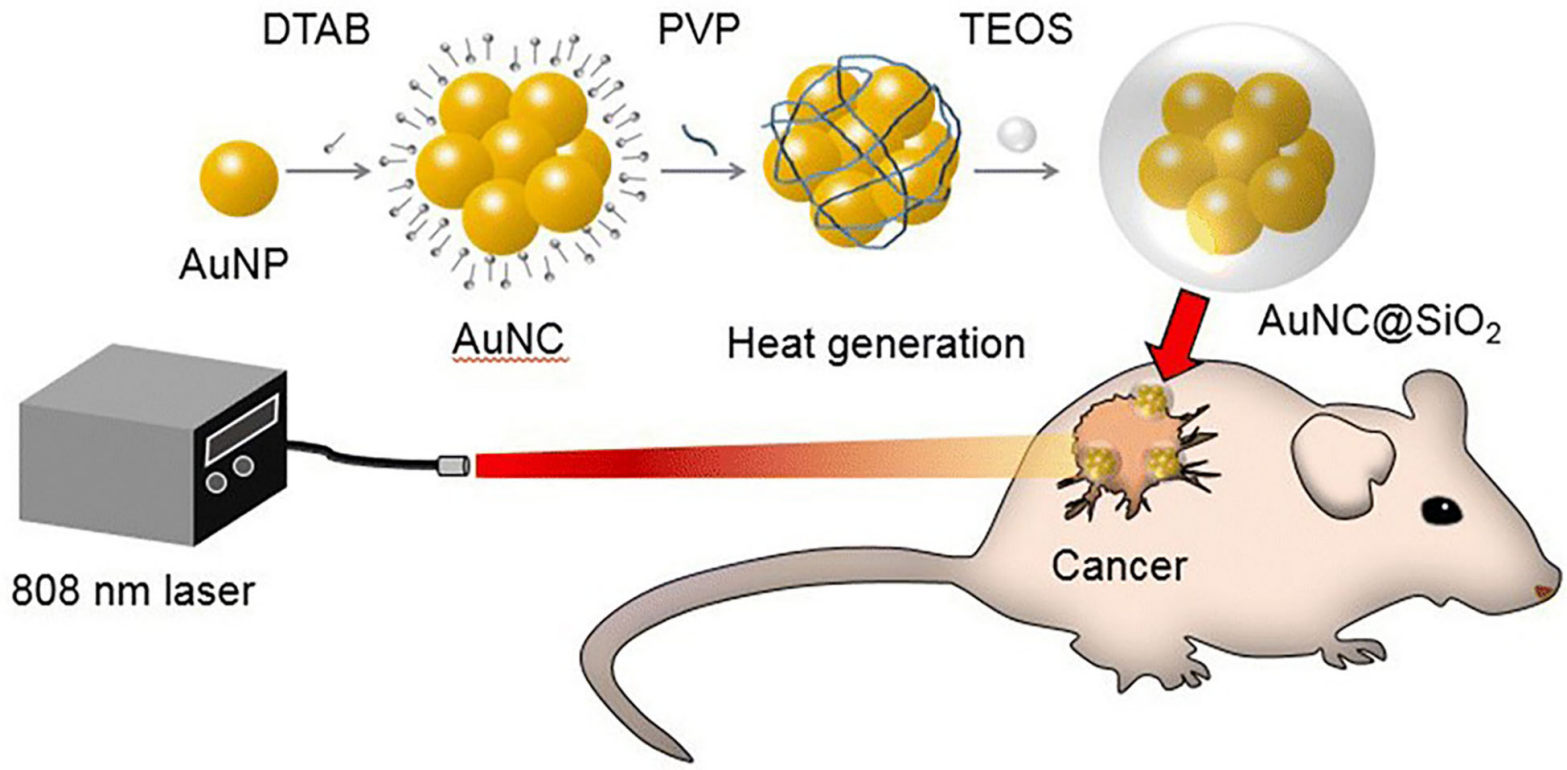
Graphical illustration showing the photothermal effect of gold nanoparticle clusters (AuNCs)@SiO_2_ in a prostate cancer model under near-infrared (NIR) laser irradiation (174).

In 2019, Rastinehad et al. conducted the first clinical pilot study of PTA in 16 prostate cancer patients, using laser-excited gold–silica nanoshells. In this experiment, NIR (810 ± 10 nm) light was used to excite gold–silica nanoshells for producing the hyperthermia condition for focal ablation of prostate cancer (173). Kim et al. demonstrated that clustering photothermal agents exhibited higher therapeutic efficiency against prostate cancer than with the single photothermal molecule. They conducted *in vitro* and *in vivo* studies with gold (Au) nanoparticles, gold nanoparticle clusters (AuNCs), and silica-coated AuNCs@SiO_2_ under the NIR laser at 808 nm wavelength against a prostate cancer model. Results showed that AuNCs@SiO_2_ possess a greater photothermal effect than AuNCs and Au nanoparticles against prostate cancer, owing to its unique nanostructure and silica layer coating, which provides stability for a long time under NIR laser irradiation (**Figure 7**) (174). Similarly, Yang et al. designed novel enzyme-triggered self-assembled gold nanoparticles with a CREKA-Y_P_ FFK(Nph) peptide sequence. Under alkaline phosphatase enzyme treatment, the CREKA-Y_P_ FFK(Nph) peptide can be self-assembled; as a result gold nanoparticles self-aggregate inside the tumor cells, and, therefore, enhance retention and photothermal effects against *in vitro* and *in vivo* prostate cancer models (175).

Target specification of tumor cells is a major concern in the modern oncology era. To overcome this, the surface of the photothermal agent can be optimized with nanopeptides for specifically targeting the cancer cells. For example, Avvakumova et al. developed neuropeptide Y (NPY)-conjugated gold nanocages and this has been exploited for localized PTA therapy against *in vitro* prostate cancer models under NIR laser treatment at a wavelength of 800 nm (176). This is because of NPY being highly expressed in prostate cancer cells and being involved in aggressive tumor growth and progression (177). In addition, Gobin et al. demonstrated that ephrinAl-conjugated gold-coated silica nanoshells have shown a potential PTA effect against a PC-3 cell line under NIR laser irradiation at a wavelength of 800 nm. This is because ephrinAl ligands easily bind to the ephrinA2 receptors, which are highly expressed on the PC-3 cell line, and thus can mediate target specificity and enhance PTA therapy (178). Therefore, targeting PCSCs with surface-functionalized photothermal agents could be a potential therapeutic treatment for prostate cancer.

## 12 Conclusions and future perspectives

Over the decades, a significant amount of research has been conducted into PCSCs and has suggested that they play a critical role in prostate cancer initiation and progression. Current conventional therapies for prostate cancer are ineffective, owing to the fact that cancer stem cells, which are not specifically targeted in most therapies, induce tumor relapse, metastasis, and therapy resistance. Therefore, the development of therapeutic strategies for targeting the PCSCs is imperative to increase treatment efficiency. miRNA-based BR-DIM and NUMB targets (Section 5), nanomedicine-based combinatorial drugs, and nanomaterials such as graphene oxide and cuprous oxide have shown promise in improving therapeutic efficacy and halting stemness features in prostate cancer stem cells. A nanotechnology platform can be adapted to target prostate cancer epithelial cells or prostatecancer stem cells by optimizing the size and shape of the nanomaterials and antibodies or nanobodies for (section 6) (72, 179), biosensor approaches (180), several enhanced chemotherapeutics agents approaches in the clinical trials and are summarized in **Tables 1**, **2** under the monotherapy and combinational therapy could be a potential strategy to employ them in the prostate cancer stem cells populations (Section 7), Immunotherapy strategies such as immune checkpoint inhibitors in the combination of core-shell nanoparticles and biomimetic nanoparticles (Section 8), CAR-T cell-based nano immunotherapeutic approaches could be more effective strategies for targeting prostate cancer stem cells for their differentiation, identification, characterization for the concomitant immunotherapeutic regime (Section 9), CRISPR-Cas nanoparticle-based strategy for understanding Yamanaka factors (section 10), different nanosystem-based hyperthermia targeting the PCSCs with photothermal ablation therapy could be potential therapeutic treatment for PCSCs for relapse by fulfilling specific markers identification, deciphering stemness-related signaling pathways and their interaction with tumor microenvironment.

## Author contributions 

SR and V-KL contributed to the conception and design of the study. SR wrote the first draft of the manuscript. PS, MA, SL, AA, SO, AM, AT, AK, YJ, SC, and V-KL finalized the second and third draft of manuscript. All authors contributed to the article and approved the submitted version.

## Glossary

**Table:**

ADT	androgen deprivation therapy
ALDH	aldehyde dehydrogenase
ASPM	abnormal spindle-like microcephaly associated
AS	active surveillance
Au	gold nanoparticles
AuNCs	gold nanoparticle clusters
BRCA1	breast cancer 1
BAZ2A-BRD	BAZ2A-bromodomain
BTF3	basic transcription factor 3
Cas9	CRISPR-associated protein 9
CAR-T	chimeric antigen receptor T
CDDP	*cis-*dichlorodiamminoplatinum
CHAARTED	Chemo Hormonal Therapy versus Androgen Ablation Randomized Trial for Extensive Disease in Prostate Cancer
CHT	chemo hormonal therapy
CRPC	castration-resistant prostate cancer
CRISPR	clustered regularly interspaced short palindromic repeats
CSCs	cancer stem cells
CUL4B	cullin 4B
DC-CIK	dendritic cells activated cytokine-induced killer
DDR	DNA damage response
DNMT1	DNA methyltransferase 1
Dvl-3	disheveled-3
E/M	epithelial and mesenchymal
EpCAM	epithelial cell adhesion molecule
ERα	estrogen receptors alpha
ERβ	estrogen receptors beta
EZH2	enhancer of Zeste homolog 2
Gli1	GLI family zinc finger 1
GO	graphene oxide
GSK3β	glycogen synthase kinase
GR	glucocorticoid receptor
GSI	γ-secretase inhibitor
HA	hyaluronic acid
HPMA	*N*-(2-hydroxypropyl)methacrylamide
HRR	homologous recombination repair
IT	immunotherapy
JAK	Janus kinase
LHRHa	luteinizing hormone-releasing hormone agonist
LNCaP	lymph node carcinoma of the prostate
mCRPC	metastatic castration-resistant prostate cancer
mHSPC	metastatic hormone-sensitive prostate cancer
miRNAs	microRNAs
mPEG-b-PCC	poly(ethylene glycol)-block-poly(2-methyl-2-carboxyl-propylene carbonate)
NIR	near-infrared
NMPC	non-metastatic prostate cancer
NPY	neuropeptide Y
OS	overall survival
PARP	poly ADP-ribose polymerase inhibitors
PBLs	peripheral blood lymphocytes
PCSCs	prostate cancer stem cells
PDGFD	platelet-derived growth factor D
PER3	period circadian regulator 3
PK	pharmacokinetics
PSCA	prostate stem cell antigen
PSCs	prostate stem cells
PTA	photothermal ablation
PTEN	phosphatase and tensin homolog deleted on chromosome 10
PTPN4	phosphatase nonreceptor type 4
RECIST	Response Evaluation Criteria in Solid Tumor
sgRNA	single guide RNA
SHH	sonic hedgehog
STAT3	signal transducer and activator of transcription 3
UCH-L1	ubiquitin C-terminal hydrolase L1
UCH-L3	ubiquitin C-terminal hydrolase L3
